# Potential for urban agriculture to support accessible and impactful undergraduate biology education

**DOI:** 10.1002/ece3.8721

**Published:** 2022-03-14

**Authors:** Adam D. Kay, Eric J. Chapman, Jelagat D. Cheruiyot, Sue Lowery, Susan R. Singer, Gaston Small, Anne M. Stone, Ray Warthen, Wendy Westbroek

**Affiliations:** ^1^ Biology Department University of St. Thomas St. Paul Minnesota USA; ^2^ 5783 Ecology and Evolutionary Biology Department Tulane University New Orleans Louisiana USA; ^3^ 7119 Biology Department University of San Diego San Diego California USA; ^4^ 8678 Office of the Provost Rollins College Winter Park Florida USA; ^5^ 8678 Social Impact Hub Rollins College Winter Park Florida USA; ^6^ Infinite Zion Farms Winter Park Florida USA; ^7^ 33065 Salish Kootenai College Pablo Montana USA; ^8^ 33065 Flathead Valley Community College Kalispell Montana USA

**Keywords:** active learning, community engagement, field course, sustainability

## Abstract

Active learning in STEM education is essential for engaging the diverse pool of scholars needed to address pressing environmental and social challenges. However, active learning formats are difficult to scale and their incorporation into STEM teaching at U.S. universities varies widely. Here, we argue that urban agriculture as a theme can significantly increase active learning in undergraduate biology education by facilitating outdoor fieldwork and community‐engaged education. We begin by reviewing benefits of field courses and community engagement activities for undergraduate biology and discuss constraints to their broader implementation. We then describe how urban agriculture can connect biology concepts to pressing global changes, provide field research opportunities, and connect students to communities. Next, we assess the extent to which urban agriculture and related themes have already been incorporated into biology‐related programs in the United States using a review of major programs, reports on how campus gardens are used, and case studies from five higher education institutions (HEIs) engaging with this issue. We found that while field experiences are fairly common in major biology programs, community engagement opportunities are rare, and urban agriculture is almost nonexistent in course descriptions. We also found that many U.S. HEIs have campus gardens, but evidence suggests that they are rarely used in biology courses. Finally, case studies of five HEIs highlight innovative programming but also significant opportunities for further implementation. Together, our results suggest that urban agriculture is rarely incorporated into undergraduate biology in the United States, but there are significant prospects for doing so. We end with recommendations for integrating urban agriculture into undergraduate biology, including the development of campus gardens, research programs, community engagement partnerships, and collaborative networks. If done with care, this integration could help students make community contributions within required coursework, and help instructors feel a greater sense of accomplishment in an era of uncertainty.

## Graphical abstract

We argue that urban agriculture is well suited as a theme for undergraduate biology education because it will facilitate the incorporation of outdoor fieldwork and community‐engaged education into the curriculum. In the manuscript, we review potential educational benefits of field courses and community engagement activities for undergraduate biology and discuss constraints to their broader implementation. We also assess the extent to which urban agriculture and related themes have already been incorporated into Biology‐related programs and make recommendations for incorporating urban agriculture into biology curricula.

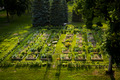

## 1 INTRODUCTION

Undergraduate attrition from science fields is a significant problem in the United States. Less than 40% of U.S. students (and ~20% of students from underrepresented groups) who start university with an interest in STEM (science, technology, engineering, and mathematics) actually graduate with an STEM degree (PCAST, 2012). This attrition contributes to a shortage of available science and health professionals and teachers (Chen, 2013; Chen et al., 2018) and represents lost investments for the students who switch majors or drop out altogether. Attrition of underrepresented students is particularly worrisome given that a diverse community of scientists reduces bias in scientific reasoning and makes science more inviting to a broader talent pool (Intemann, 2009; Sulik et al., 2021). Moreover, selective attrition negatively impacts classroom dynamics by reducing the range of identities and backgrounds contributing to the educational process.

One reason identified for STEM attrition is because science content and class activities can seem inaccessible and lack relevance, especially for first‐generation college students from a variety of economic, racial, and ethnic backgrounds (Estrada et al., 2016; Seymour & Hunter, 2019). Concepts in biology involve complex processes at scales that can be difficult to perceive. Furthermore, introductory laboratories are often built around easy‐to‐follow procedures where instructors know what the results will be, leaving students with the impression that biology—and more generally, the scientific method—involves following established protocols and measuring expected outcomes. In addition, STEM instruction rarely connects to pressing social challenges that many students are keenly aware of.

Recent summaries of education research have suggested ways to address these shortcomings. The 2011 American Association for the Advancement of Science's (AAAS) *Vision and Change* report outlined guidelines to improve undergraduate biology education and highlighted core concepts and competencies required for modern biologists (AAAS, 2011, Figure 1). It emphasized integrating these core concepts and competencies throughout the curriculum and focusing on students as active participants in the educational process, among other recommendations. To help students develop this conceptual understanding and these competencies, programs began moving from lecture to active learning formats (Freeman et al., 2014) and created course‐ and non‐course‐based undergraduate research experiences (Banger & Brownell, 2014). Evidence suggests that this movement has had particular benefits for STEM students from underrepresented backgrounds and for female students in male‐dominated fields (Haak et al., 2011; Odom et al., 2021). Subsequent reports have emphasized the overwhelming evidence from educational research about the benefits of active learning innovations that have increased student engagement by creating student‐centered, inquiry‐rich experiences (Laursen, 2019).

**FIGURE 1:**
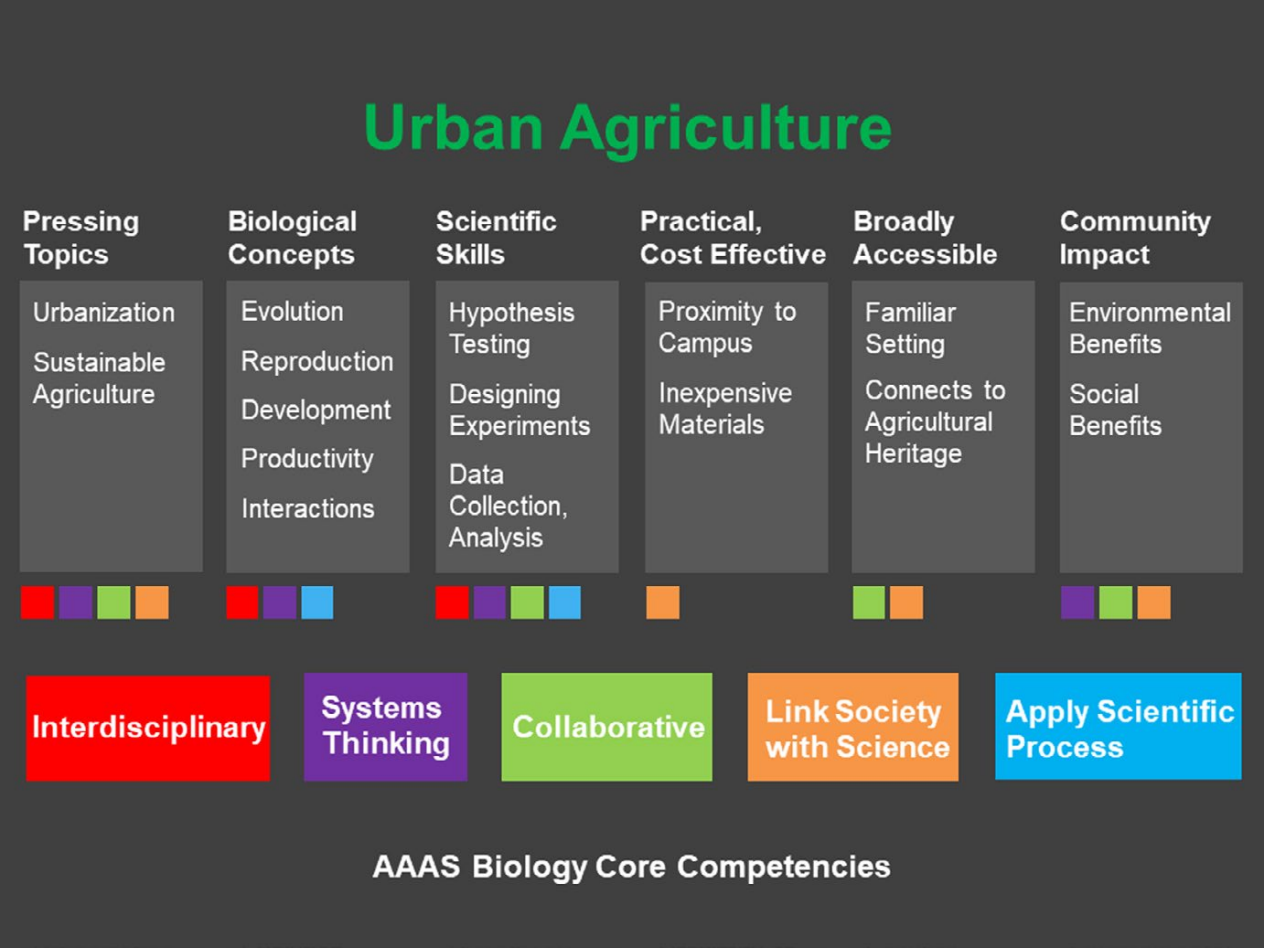
Conceptual framework linking urban agriculture to biology curriculum grounded by the AAAS Vision and Change report (AAAS, 2011)

However, despite their educational benefits, immersive, active learning formats are often difficult to scale and the extent to which they have been incorporated into STEM teaching at U.S. universities varies widely (Laursen, 2019; Nguyen et al., 2021; Stains et al., 2018). Two approaches for scaling up active learning in biology involve the incorporation of outdoor fieldwork (Easton & Gilburn, 2012) and the use of community‐engaged education (Hansen et al., 2021). Although both approaches have been shown to improve learning outcomes in undergraduate students, both face significant barriers to broad implementation in biology (described more thoroughly below).

Here we argue that urban agriculture, which includes everything from backyard urban gardening to large‐scale food production operations in cities (Wortman & Lovell, 2013), has a largely untapped potential to integrate field experiences and community‐engaged education into undergraduate biology education. Urban agriculture can provide active learning, research‐intensive educational experience in a way that can be practically applied to a large number of students. At the same time, development of undergraduate biology experiences around urban agriculture can help biology departments and their universities develop lasting and mutually beneficial engagements with community organizations that can in turn inspire student learning.

We begin with an overview of potential educational benefits of fieldwork and community engagement for undergraduate biology and discuss constraints to implementation. We then describe how urban agriculture programs can help to overcome these constraints. We assess the extent to which urban agriculture has been incorporated into undergraduate biology curricula using both a review of major programs and an analysis of a database from the American Association for Sustainability in Higher Education (AASHE) and present case studies of urban agriculture activities in bioscience departments at five higher education institutions (HEIs). Finally, we make recommendations for how to incorporate urban agriculture into undergraduate biology education. Given our collective expertise, we focus our discussion on HEIs in the United States, but we hope that it can help with reform in undergraduate biology education in other countries as well. While our presentation in some ways parallels calls for the development of “sustainable food systems education” aimed at future professionals (e.g., Sterling et al., 2021), we suggest instead that incorporating urban agriculture into undergraduate biology will benefit all biology students, regardless of career interests, by engaging them in basic science and exposing them to pressing community challenges.

## 2 BENEFITS AND CHALLENGES TO FIELD‐BASED AND COMMUNITY‐ORIENTED BIOLOGY EDUCATION

Although field (outdoor) experiences have long been viewed as essential to biology education, recent reviews have documented the decline in field‐oriented courses in U.S. higher education (Easton & Gilburn, 2012; Fleischner et al., 2017). Field courses can engage students in active, research‐oriented learning, help them gain environmental knowledge, and inspire them to social responsibility around sustainability issues. These experiences can help students integrate knowledge and achieve higher‐order learning (Durrant & Hartman, 2015; Easton & Gilburn, 2012) and can reduce achievement gaps correlated with gender and racial identity and socioeconomic status (Beltran et al., 2020). Despite these benefits, field experiences are becoming increasingly limited because of the increasing institutional focus on liability issues and the financial and time requirements for field experience development (Fleischner et al., 2017). Although the Undergraduate Field Experiences Research Network (https://ufern.net/) is increasing field experiences at field and marine stations, these experiences will likely still be out of reach for many students.

Community‐engaged education has similarly received increased emphasis due to the growing awareness that HIEs must produce graduates who have the awareness to address pressing social challenges (Hansen et al., 2021). Multiple methods have emerged to increase community engagement for HEI students, including service learning, community‐engaged learning, and community outreach programs (Schatteman, 2014), and this engagement has been extended further through the emergence of citizen science (NASEM, 2018). These educational efforts build off findings that many students are motivated to help others (Gorski et al., 2015), and community‐oriented experiences have been shown to pique students’ interests, enhance learning outcomes, improve retention of female‐identifying students in STEM, and lead to more students expressing interest in pursuing service‐related career opportunities after college (Diekman et al., 2015; Mehta et al., 2015; Ryan, 2017; Tannenbaum & Berrett, 2005). Although community‐engaged education has the potential to be incorporated into STEM education as a complement to content‐oriented coursework and on‐campus environmental activism, its incorporation into U.S. undergraduate biology education is rare (Zizka et al., 2021, and see below, but see Marx et al., 2021, Nation & Hansen, 2021, Yep et al., 2021). Barriers to broader implementation include resources, time, or experience to develop connections with community partners, challenges in obtaining institutional support (Mehta et al., 2015), and frameworks to help students connect theoretical classroom content to application in communities (Zizka et al., 2021).

Urban agriculture has the potential to expand field‐oriented and community‐engaged education into undergraduate biology programs in a way that is both practical and impactful, especially for students on urban campuses. Urban agriculture experiences can provide field course experiences on or near campuses (see below), overcoming the time and resource requirements that are significant barriers to implementing field courses that serve large numbers of students. Urban agriculture can also allow for the application and extension of traditional biology theory in diverse subdisciplines. Finally, urban agriculture is at the core of many community engagement/service‐learning experiences in other disciplines, and introducing a “community‐oriented” urban agriculture into biology courses should provide new ways for biology departments to help students and faculty connect their activities to social challenges.

## 3 POTENTIAL RECIPROCAL RELATIONSHIP OF URBAN AGRICULTURE AND UNDERGRADUATE BIOLOGY EDUCATION

Urban agriculture has rapidly expanded in North America through the development of community gardens and small farms (Fox, 2018; Rizzo, 2021). Although some commercial urban agriculture has arisen, urban agriculture's expansion has been driven primarily by an idealism emerging from a variety of social, environmental, and public health benefits (Nogeire‐McRae et al., 2018).

This ideologically based emergence suggests that urban agriculture as a theme can align well with core concepts and competencies highlighted in Vision and Change (Figure 1). First, the topic serves as a connection between two major global trends, the rapid expansion of urbanization (Elmqvist et al., 2019; Seto et al., 2011) and the economic and environmental challenges facing global agriculture (Foley et al., 2011; Rockström et al., 2017). Second, urban agriculture makes biology concepts more apparent for students. Production and other crop features are easy‐to‐visualize outcomes that can make biological concepts, such as adaptation, reproduction, development, productivity, and interactions, tangible across various scales of organization. This tangibility can benefit biology majors but may be particularly relevant for nonmajors with limited exposure to instruction on the process of science. Third, urban agriculture's small scale creates opportunities for replication across gardens or plots within gardens, making it well suited for teaching experimental design, interactions, and systems thinking. Fourth, urban agriculture is practical; sites can be on or close to campus and supplies are relatively inexpensive. Fifth, urban agriculture has many social benefits, including foregrounding indigenous knowledge and agricultural heritage and helping to build connections among diverse communities. And sixth, urban agriculture and more generally food systems as a theme can connect undergraduate biology to chronic disease and other human health challenges that are particularly engaging for the large number of undergraduate biology majors interested in health care as a career. Together, these features of urban agriculture suggest that its use in biology education can engage students by connecting them to local and global challenges that resonate with them.

Below, we describe the present state of and future opportunities for urban agriculture in undergraduate biology. First, we survey top‐rated research and liberal arts colleges to describe whether and how food systems, community engagement, and, more specifically, urban agriculture are currently incorporated into biology curricula. Second, we assess how campus gardens are being used in undergraduate teaching and research to determine the extent to which these gardens could help biologists incorporate urban agriculture into their courses. Third, we present brief case studies focused on biology programs at five universities and colleges to identify opportunities for further incorporation of urban agriculture. We end with some suggestions for future expansion and for overcoming barriers to implementation.

## 4 URBAN AGRICULTURE IN THE CURRICULUM—CURRENT STATE

We used three types of information to assess the current extent to which urban agriculture has been incorporated into undergraduate biology education in the United States: a review of major programs, an assessment using a database from AASHE, and case studies from developing programs. Although each assessment type has limitations, collectively they provide information about opportunities and constraints on the widescale development of the approach we are highlighting.

### 4.1 Current presence of urban agriculture and related themes in major HEI biology programs

In October 2021, we surveyed online presentations of biology or biology‐related programs in 40 top‐rated research and liberal arts HEIs to assess the extent to which community‐oriented urban agriculture has been incorporated into U.S. biology curricula. We identified HEIs for this survey using U.S. News Reports. Although we are agnostic regarding the educational value of these reports, we focus on them as a way to identify programs that are highlighted for their potential value to prospective students.

We used publicly available course catalog information from each institution to conduct the survey. We surveyed catalogs to find descriptions of all courses with a BIOL or equivalent course label at each institution. We first sought to identify titles and descriptions for content focused on urban agriculture and related themes. Specifically, we searched for (1) “food,” “agricultur,” or “agro,” (2) “urban,” and (3) “urban agriculture” and screened all positive returns to determine if courses were associated with human food systems, urban areas, and urban agriculture, respectively. Then, we searched for courses that advertised field experiences and community engagement activities—main general benefits of urban agriculture—to determine the extent to which these benefits are already present in undergraduate biology curricula. Specifically, we searched course titles and descriptions for (1) “field” and (2) “communit,” “service,” or “experiential” and screened all positive returns to identify courses that advertised field experiences and community engagement activities, respectively.

Our survey reveals several interesting findings about urban agriculture, field biology, and community engagement in undergraduate biology (Table 1, Table S1A,B). First, food systems and, to a lesser extent, urban systems are represented in biology curricula at top‐rated schools, but urban agriculture as a theme is essentially nonexistent. On average at the time of our survey, research HEIs offered 2.55 ± 0.72 (mean ± SE) courses that mentioned food systems‐related terms and 0.15 ± 0.11 courses that mentioned urban systems‐related terms in course descriptions. Our surveyed liberal arts HEIs offered 1.55 ± 0.43 courses mentioning food systems; one example was BIOL 225 Sustainable Food and Agriculture (Williams College). We also found that 0.35 ± 0.11 course descriptions for our surveyed liberal arts HEIs mentioned urban issues; an example is BIOL 330 Urban Ecology and Evolution (University of Richmond). Only one course in the survey mentioned urban agriculture: BIOEE 4690 Food, Agriculture, and Society (Cornell).

**TABLE 1:** Summary information from course descriptions in Biology‐related departments in top‐ranked research HEIs (*n* = 20) and liberal arts HEIs (*n* = 20)

Institution type	Search term(s)				
	“field” (related to field experiences for students)	“communit” OR “service” OR “experientia” (related to community‐engaged learning)	“food” OR “agricultur” OR “agro” (related to food systems)	“urban” (related to urban systems)	“urban agriculture”
Research HEIs	10.5 ± 2.34 (range: 0–34)	0.45 ± 0.17 (range: 0–3)	2.55 ± 0.72 (range: 0–11)	0.15 ± 0.11 (range: 0–2)	0.05 ± 0.05 (range: 0–1)
Liberal arts HEIs	5.20 ± 0.66 (range: 0–11)	0.20 ± 0.09 (range: 0–1)	1.55 ± 0.43 (range: 0–7)	0.35 ± 0.11 (range: 0–1)	0

Note

Data are mean + SE number of courses for which select terms are mentioned in course titles or descriptions. More detailed information is in Table S1A,B.

Main potential benefits from urban agriculture—field experiences and community‐engaged learning—were differentially represented in the survey. Field courses were well represented in course descriptions for top‐rated programs, particularly at research institutions. On average, research HEIs offered 10.5 ± 2.34 (mean ± SE) courses that advertised a field component (range 0–34) and liberal arts HEIs offered 5.2 ± 0.66 courses (range 0–11). Field courses generally emphasized experience in areas of low human impacts (e.g., field stations, natural preserves). In contrast, courses advertising community engagement opportunities were rare. Only 7 of 20 biology‐related programs at research HEIs had any courses mentioning community engagement (mean # courses ± SE = 0.45 ± 0.17) and only 4 of 20 at liberal arts HEIs mentioned community engagement (mean # courses ± SE = 0.20 ± 0.09). Examples of biology‐related courses mentioning community engagement are BIOL 0371 Advanced Field Biology: Place‐based Global Biology Education (Middlebury) and BIOL 036 Ecology (Swarthmore), which describes “collaboration with local stakeholders and engagement with both Indigenous and Western approaches to understanding humans’ connection with the natural world….”

There are caveats when considering the results of this survey. Instructors may incorporate modules and themes into courses without mentioning them in course descriptions, which are often generic and general to provide flexibility for instructors seeking to deliver dynamic content. In addition, many schools give faculty opportunities to offer “topics courses” that are more experimental and specific. These courses are only reviewed by college‐ or university‐level curriculum committees after several iterations. As a result, our survey is likely missing course content and projects that are related to urban food systems and communities. Most importantly, our survey of “top‐rated” schools is not a random sample of U.S. HEIs and thus unlikely represents accurately the challenges facing undergraduate biology education nor the extent to which creative solutions are emerging to address those challenges. In particular, the presence of field experiences in pristine locations is likely overrepresented in well‐resourced schools given the costs of these experiences. In addition, it is possible that “urban” as a theme is underrepresented in top‐rated liberal arts colleges in particular given that many of these schools are in nonurban settings.

Regardless, we draw several conclusions from these results. First, food systems and urbanization as themes are not common in undergraduate biology curricula, at least in our surveyed institutions. There are several reasons for this scarcity, including that these themes may be well represented in other departments or schools at the institution. Regardless, this rarity suggests that undergraduate biology has the opportunity to adjust curricular offerings to help students address pressing global challenges. Second, urban agriculture as a theme is essentially nonexistent in biology programs at top‐rated schools. Finally, field experiences, a main potential benefit of urban agriculture, are fairly common, but it is unclear how widespread and costly these experiences currently are. Another main benefit of urban agriculture for biology—community engagement—is very rare in these programs. Together, these results suggest that there is a significant opportunity for expansion of urban agriculture and related themes in undergraduate biology.

### 4.2 Campus gardens in undergraduate biology education?

Another source of information about urban agriculture in biology education is assessing the extent to which campus gardens are incorporated into biology curricula. Food gardens on HEI campuses have become much more common over the past few decades, and research has shown that they can provide diverse benefits to students, faculty, institutions, and surrounding communities (Marsh et al., 2020). For students and faculty, campus gardens can provide field‐based teaching and research opportunities across diverse disciplines (Scoggins, 2010, and see our review below), contribute positively to student mental health (Cupples & Finewood, 2018), and increase student access to organic foods (Ullevig et al., 2020). For institutions, campus gardens are often part of sustainability plans and are used in recruitment and outreach efforts to prospective students and donors (Duram & Klein, 2015; Duram & Williams, 2015; Laycock Pedersen & Robinson, 2018). For communities, campus gardens can support outreach events (Anderson et al., 2018) and inspire students to become involved in service activities.

Here, we compiled data from AASHE Sustainability Tracking and Rating System (STARS) database (https://stars.aashe.org) to determine the extent to which U.S. HEIs have campus gardens and how commonly gardens are incorporated into teaching and research in biology and related disciplines. Although several case studies have examined the impacts of campus gardens on student engagement, experiential learning, and campus sustainability, there is scant research on the extent to which gardens are incorporated into educational programming in the U.S. HEIs in general. One example is Duram and Klein (2015), which surveyed 52 campus gardens in the United States and found that 92% of garden manager respondents cited “education” as the primary goal of the garden and indicated that course experiences were primarily in the areas of sustainability and environmental studies. To our knowledge, there is no information about incorporation of campus gardens into biology‐related programs.

We included in our survey results from 286 institutions of higher education that submitted AASHE reports in 2019. We compiled information on campus gardens or farms from several sections of the report (Table S2). For all institutions that reported having a campus garden or farm, we searched the descriptions in the other fields in Table S2 for (1) any explicit mention of using campus gardens/farms in formal undergraduate or graduate classes, and (2) any explicit mention of using campus gardens/farms for research. Class‐integrated research projects were counted in both categories. We also searched websites of campus gardens/farms reported in the STARS report for any explicit mention of use by courses or research. Where specific courses were listed, we categorized those courses as either Biology, Environmental Studies/ Sustainability, Horticulture, Nutrition, or Other. We then determined the percentage of institutions from each institution type (Associate, Baccalaureate, Master, Doctoral/Research) that reported having a campus garden or farm project, the percentage of those institutions (of each institution type) that reported (either in the STARS report or on the website) using the project in teaching or in research, and the courses listed as using the project.

Almost all institutions in the STARS database reported that they were associated with some type of garden or farm. In response to the question *“Does the institution have gardens*, *farms*, *community supported agriculture (CSA) or fishery programs*, *and*/*or urban agriculture projects where students are able to gain experience in organic agriculture and sustainable food systems?”*, 270 of 286 institutions (94%) answered “yes.” Almost all institutions responded positively regardless of institution type (Associate college: 11/12 (92%), Baccalaureate: 73/75 (97%), Masters: 65/72 (90%), Doctoral/Research: 119/125 (95%), Other: 2/2 (100%)).

Of the HEIs that reported having agriculture‐ or urban agriculture‐related projects, we found that many (119/270 = 44%) indicated that projects were used for teaching or research. Percentage use in teaching or research did not differ significantly across institution type (Associate college: 5/12 (45%), Baccalaureate: 38/73 (52%), Masters: 30/65 (46%), Doctoral/Research: 46/119 (46%), Other: 0/2 (0%)). For HEIs that listed specific courses using campus gardens/farms, we found that the majority of courses were in either Environmental Studies or Sustainability (52%), and 26% were in Biology (Table 2). Together, these results indicate that many HEIs in the United State have urban agriculture‐type projects, but less than 10% of all institutions reporting these projects indicated that the projects were used in biology courses.

**TABLE 2:** Information from American Association for Sustainability in Higher Education (AASHE) Sustainability Tracking and Assessment System (STARS) reports and website scans about the topic of courses that use campus farms/gardens in teaching

Institution type	Course subject					
	Biology	Environmental studies/sustainability	Horticulture	Nutrition	Other	Total
Associate	1	0	2	0	0	3
Baccalaureate	12	16	1	0	4	33
Master	4	17	3	0	1	25
Doctoral/Research	7	16	3	3	4	33
Total	24 (26%)	49 (52%)	9 (10%)	3 (3%)	9 (10%)	94

It is important to note that institutions completing AASHE STARS reports likely disproportionally invest in sustainability‐related projects, suggesting that the true number of U.S. HEIs with urban agriculture‐type projects and the extent to which biology courses are connected to such projects are likely less than reported here. Also, the most relevant STARS report question asks only if teaching OR research is connected to urban agriculture‐type projects, making it difficult to assess precisely how such projects are connected to the curriculum.

Regardless of these caveats, these results suggest that urban agriculture‐related projects are very common at HEIs, and there is significant opportunity for expanding their use in undergraduate biology education.

### 4.3 Case studies

Our case studies provide additional context and ideas about how urban agriculture might be incorporated into undergraduate biology. We focus on five institutions represented by the authors—University of St. Thomas (MN), Tulane University (LA), Salish Kootenai College (MT), University of San Diego (CA), and Rollins College (FL). These institutions were first identified because of their involvement in the Ashoka Changemaker Campus network, an international effort that brings together institutions committed to connecting higher education to community engagement and social innovation. These institutions have also partnered to develop a Research Collaboration Network sponsored by the Undergraduate Biology Education program of the National Science Foundation (NSF‐UBE).

#### 4.3.1 Institution

University of St. Thomas (UST)—Biology Department.

##### Institutional overview

The UST Biology Department offers two lower‐division and three upper‐division elective courses focused on food systems. Of the lower‐division courses, BIOL 209 Biology of Sustainability is a major‐required core course with a 3‐week unit focused on global agricultural challenges, and BIOL 296 Future of Food is an elective 2‐credit seminar focused on readings and discussions about current innovations and constraints on food production, distribution, and consumption. Part of the seminar includes site visits and guest lectures by local practitioners. Of the upper‐division elective courses, BIOL 315 Plants, Food, and Medicine is a modified plant physiology course that has incorporated sustainable food production as a theme. BIOL 490 Sustainable Food Systems is a senior seminar capstone that includes semester‐long small‐group projects completed in collaboration with a variety of community partners. Examples of student‐led projects include an investigation of food insecurity on and off campus and an assessment of perceptions of conservation agriculture in rural communities using interviews and surveys. BIOL 498 Urban Agriculture and Social Innovation in Cape Town, South Africa is a study abroad course that focuses on ecology‐oriented student projects completed with farmers in community gardens in the Khayelitsha township. Activities in this course are coordinated with Abalimi Bezakhaya (abalimibezekhaya.org.za), a nonprofit micro‐farming organization focused on assisting impoverished groups and communities in the Greater Cape Town area.

##### Biology co‐curriculum related to urban agriculture

**FIGURE 2:**
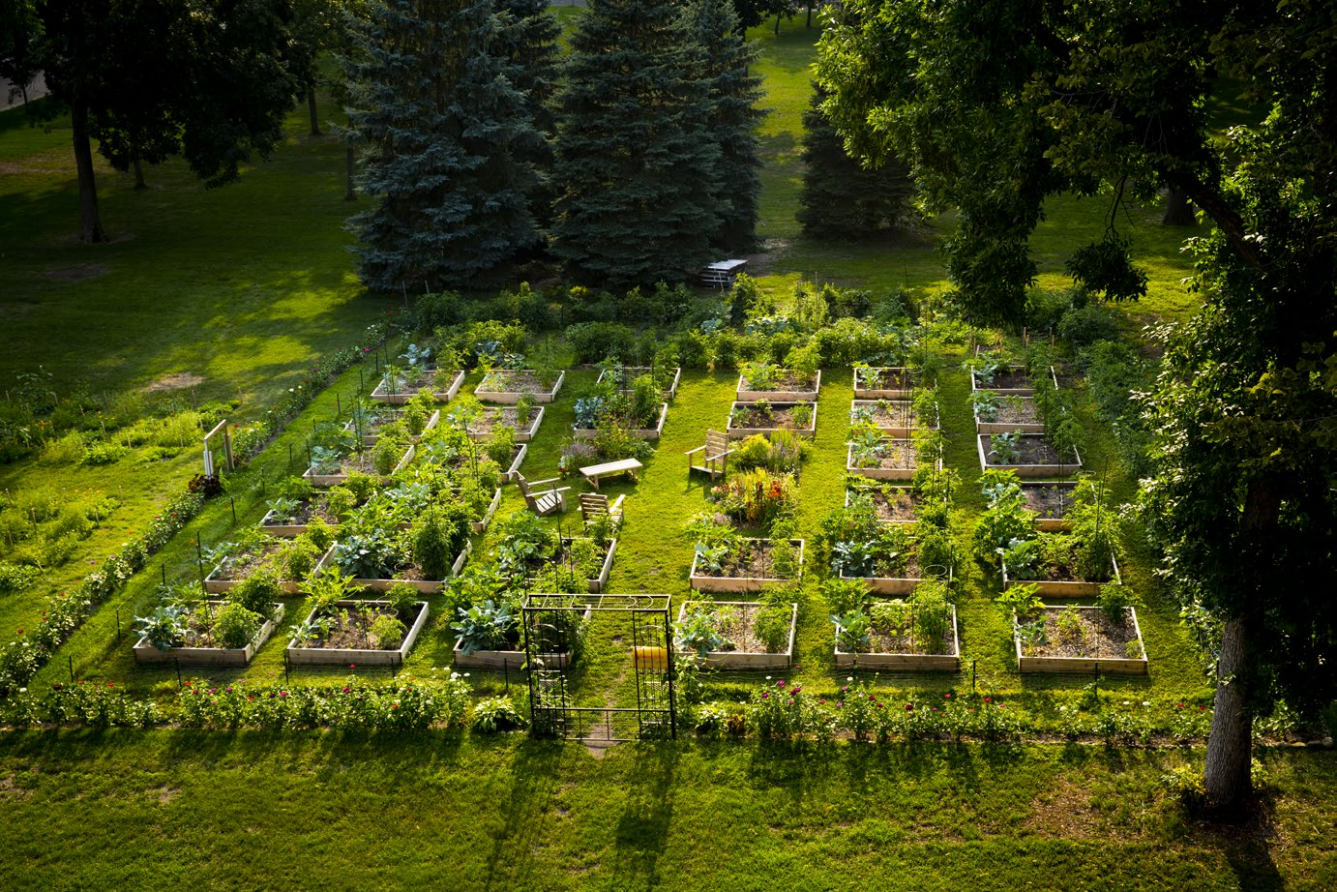
The Stewardship Garden at the University of St. Thomas (MN) illustrates how campus gardens can be used in biology teaching and research (photo: B. Brown)

The emergence of food systems‐related curriculum has coincided with the development of an on‐campus community garden project, the UST Stewardship Garden (SG) (Figure 2). This project was developed in 2010 to combine undergraduate‐driven research, teaching, and community engagement around the theme of urban agriculture. The SG consists of community garden–style plots embedded in an on‐campus gathering space. As a research resource, the SG has supported the work of over 50 undergraduate and high school researchers on questions relevant to local agriculture communities and the broader fields of ecology and environmental science. Data from the site have contributed to eight peer‐reviewed science publications with undergraduate authors and a major research grant from the NSF. Examples of projects include evaluating alternative composting processes (Small et al., 2017) and quantifying nutrient loss and recovery from different fertilization and planting strategies (Shrestha et al., 2020). As a teaching resource, the SG has provided tangible and accessible opportunities for developing core STEM competencies, including systems thinking, engaging broadly with the scientific process, and linking science to society (Figure 1), through experiences in nonmajors, core, and upper‐division courses, while helping students make connections between STEM, the liberal arts, and social/environmental challenges. These educational benefits led to the development of the NSF‐UBE network. Finally, as a community engagement resource, the SG site has produced ~2,000 lbs of produce donations each year; hosted workshops and other gatherings with campus members and the general public; and spawned a nonprofit (BrightSide Produce) that distributes fruits and vegetables to corner stores in underserved urban neighborhoods.

##### Opportunities for growth at St Thomas

Connecting activities in the Biology Department to the thriving local food system movement in the Twin Cities could enhance student experiences in the courses listed above and other existing classes. Providing more opportunities for long‐term relationship building with community members could help students build empathy and understanding. Expanding international experiences like BIOL 498 Urban Agriculture and Social Innovation in Cape Town, South Africa would provide students with opportunities to connect to marginalized communities in more authentic and less transactional experiences. Opportunities also exist for collaborating with other disciplines within the institution to create more impactful projects.

#### 4.3.2 Institution

Tulane University—Ecology and Evolutionary Biology (EEB).

##### Institutional overview

The primary campus of Tulane is located in uptown New Orleans and occupies 130 acres. Tulane is well known for its investment in community engagement and service learning, and students provide more than 750,000 hours of community service each year.

##### Biology curriculum related to urban agriculture

Tulane's Ecology and Evolutionary Biology (EEB) Department has five major courses with a connection to food systems: (1) EBIO 2210: Insect and Human Interaction has a module in which students study insect behavior and interactions in urban gardens. (2) EBIO 3180: Plants and Human Affairs focuses on the interaction between humans and plants with an emphasis on food security in New Orleans and globally. Community gardens are used in plant propagation projects. This course attracts students from different disciplines including business and political science students. Goals for this class are to engage such students to work on collaborative, interdisciplinary projects with community organizations addressing food insecurity in New Orleans. (3) EBIO 3590: Plant Biology and Adaptation uses an urban garden to study plant growth, propagations, and plant–insect interactions. (4) In EBIO 4430: Entomology, students use local settings to design experiments to study vegetable and ornamental plant pests. Finally, (5) EBIO 4600 Urban Agroecology and Sustainability in New Orleans is a project‐centered course in which students evaluate ecological interactions and use their knowledge to increase produce productivity in urban gardens.

##### Biology co‐curriculum related to urban agriculture

Through service learning, Tulane has developed gardens in two neighborhoods in the city. The service‐learning courses are optional components in some EEB courses. Two gardens have been developed in partnership with the Mardi Gras Indians Chiefs Council and New Zion Baptist Church, and two others have been developed in the Broadmoor neighborhood through a partnership with the Broadmoor Improvement Association.

##### Opportunities at Tulane

Developing an on‐campus community garden to complement Tulane's off‐campus urban agriculture would create opportunities for conversations among Tulane community members about fresh produce access and increase opportunities for interdisciplinary collaboration (with Economics, Sociology, and other disciplines).

#### 4.3.3 Institution

Salish Kootenai College—Biology.

##### Institutional overview

Salish Kootenai College (SKC) is a Tribal College chartered by the Confederated Salish and Kootenai Tribes. The campus is 72 acres and is located on the rural Flathead reservation in Montana. SKC serves about 700 students representing 68 North American Tribes, and 68% of the students are first‐generation college students.

##### Biology (Life Sciences) curriculum related to urban agriculture

SKC Life Sciences department is the only 4‐year biomedically‐oriented Life Sciences program at a tribal college nationwide. A majority of students taking biology courses have an interest in a career in health care such as nursing. The department offers Cellular Biology and Environmental Health tracks. Courses include biology standards (e.g., Cellular Biology, Genetics, Bioinformatics) with some specific environmental health offerings (Virology, One Health, Environmental Toxicology). Currently, no courses are specifically linked to urban agriculture or, more generally, to food systems or urbanization.

##### Biology (Life Sciences) co‐curriculum related to urban agriculture

Currently, SKC has a community greenhouse and garden, both part of the SKC extension initiative. The maintenance is done by volunteers, both students and community members, and students with paid positions. The extension initiative is mainly funded through federal grants from the U.S. Department of Agriculture (USDA), NSF, and National Institute of Health (NIH‐INBRE). Each Fall, the community garden runs a 10‐week sustainable diet program in which 40 community members can enroll for curriculum on sustainable healthy diets.

##### Opportunities for growth at SKC

Integration of the community garden in Life Sciences program could create opportunities for cross talk between classes spanning the curriculum. The community garden could also create entrepreneurship experiences and a closer collaboration with the SKC Business department. Finally, there is untapped opportunity for involvement of Life Sciences faculty and students with the 10‐week sustainable diet SKC extension program to include biomedical research on health disparities in Native American communities.

#### 4.3.4 Institution

University of San Diego—Biology.

##### Institutional overview

USD is a comprehensive Catholic university with approximately 9000 students. It is situated in a diverse urban community less than 20 miles from an international border (Mexico). The student body comprises 37% minority and 9% international students. Community service and change‐making are institutional priorities.

##### Biology curriculum related to urban agriculture

The Biology Department at USD emphasizes undergraduate research, requiring every student to engage in an independent research project with a USD faculty mentor, a course‐based research experience, or an off‐campus research internship. Students in BIOL 240 Bioenergetics and Systems (approximately 170–190 students per semester) conduct greenhouse experiments to compare effects of commercial versus organic fertilizer on tomato plant growth and physiology. Students in BIOL 472 Plant Physiology conduct a variety of experiments with agricultural crop species, including plant cell culture for producing clonal lines, plant responses to herbivory, and plant growth regulators. Typically, at least one section annually of BIOL 309 Research Methods uses agricultural crop species as the experimental system. Finally, students in BIOL 113 Plants and People for nonmajors conduct experiments with commercial crop species, visit Wild Willow Farms (a local urban educational agriculture facility), and engage in community service‐learning activities in school gardens at a local middle school.

##### Biology co‐curriculum related to urban agriculture

USD has a community garden established and maintained by student volunteers. Currently, there are no connections between the biology curriculum (or any other curriculum) and the student garden.

##### Opportunities for growth at USD

Enhancing the framework of sustainable agriculture and healthy food systems to introductory courses would allow biology students to integrate biology, social justice, sustainability, and climate change more explicitly. Converting the BIOL 240 Bioenergetics and Systems tomato plant experiment from a greenhouse to a community garden project could create a more expansive and integrative learning experience and help to the ecological and human health benefits of urban gardening. In particular, leveraging the health benefits of urban gardening would promote more buy‐in from students interested in health professions. Conducting biology research in the community garden could serve a large number of summer research students or those engaged in a course‐based research project. Other opportunities include connecting biology experiences in urban agriculture to living‐learning community (LLC) initiatives that integrate social and academic experiences around themes such as Advocate, Cultivate, and Innovate. Finally, expanding the exploration of urban agriculture and links to diet impacts on health, food security issues, and environmental sustainability are potentially fruitful ways to connect the biology curriculum to change‐making and community engagement—a central part of the USD mission.

#### 4.3.5 Institution

Rollins College—Biology.

##### Institutional overview

Rollins College is a co‐educational liberal arts college just north of Orlando, FL. It has approximately 3300 students. The institution website emphasizes that students will be able to “connect [their] education and … passions to the needs of the world.”

##### Biology curriculum and co‐curriculum related to urban agriculture

**FIGURE 3:**
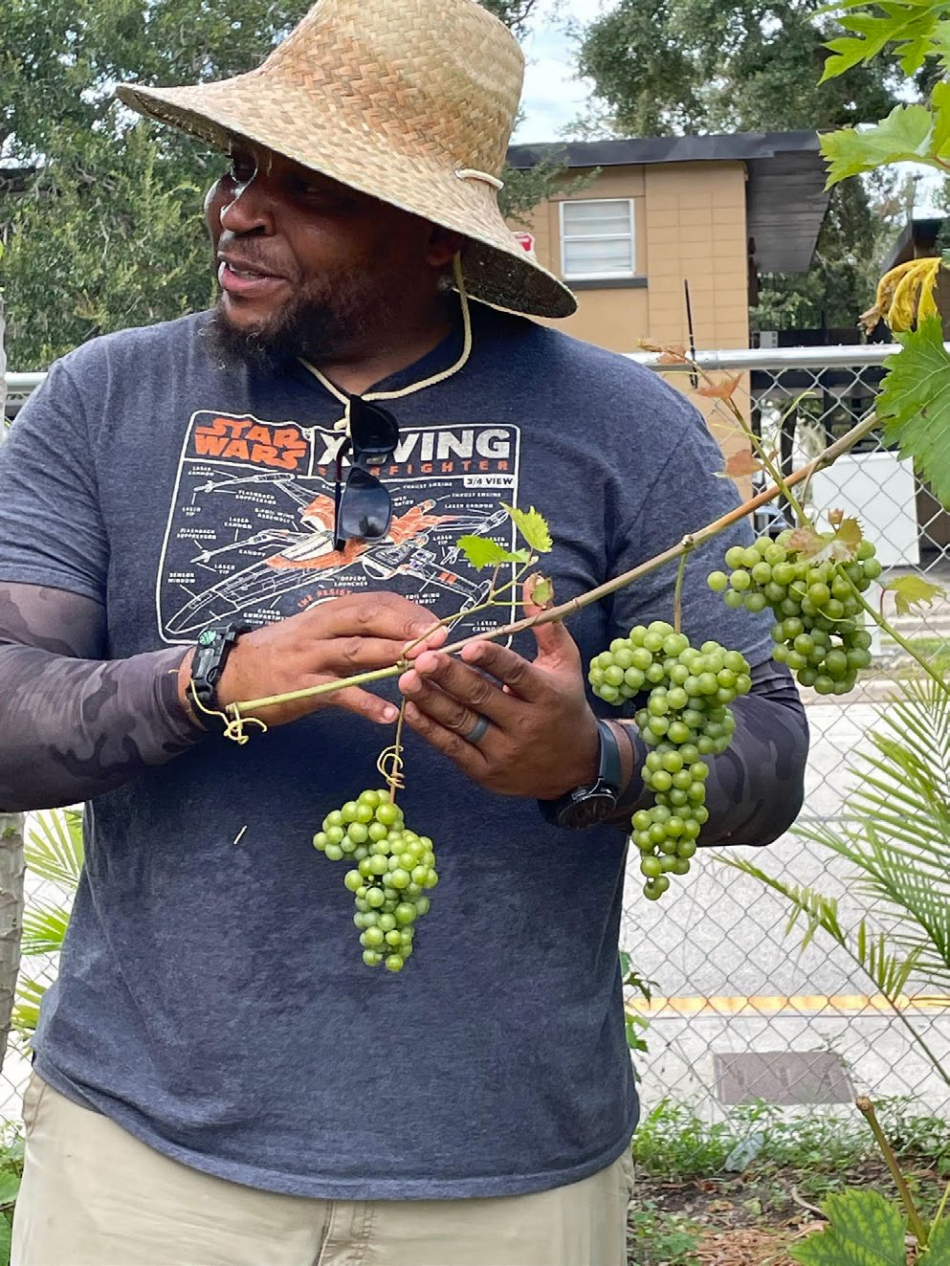
Ray Warthen of Infinite Zion Farms in a vacant lot farm in Orlando, FL (photo: E. Chapman)

The Biology Department is exploring potential connections to urban agriculture on campus and in the community. Rollins has a campus garden “the Urban Farm” that grew over the last 5 years from a student endeavor to a collaborative effort among students, faculty, and staff. This garden is now central to a Sustainable Agriculture course offered through Environmental Studies. Biology is collaborating with Rollins’ Social Impact Hub, a creative space for human‐centered design thinking, to build connections with urban agriculture organizations in Orlando. These organizations include Infinite Zion Farms (Figure 3), which establishes farms in the Parramore neighborhood of Orlando and elsewhere as an affordable source of organic produce and education to the community, and 4roots, a community alliance seeking to build a sustainable food system.

##### Opportunities for growth at Rollins

For Rollins, key next steps involve more fully integrating the Urban Farm work into the Biology major, both in plant biology and ecology. Collaborations with the campus food service are underway to determine which crops, during which season, are most likely to enhance the value of the joint effort to bring campus‐grown food to students’ tables. Further, comparative experiments in hydroponic gardening between the Rollins’ Urban Farm and Infinite Zion Farms are about to be launched. The opening of a new research greenhouse on the campus freed up a hoop house greenhouse adjoining the Urban Farm that will extend the gardening season into the brief winter months in Florida.

## 5 NEXT STEPS AND GENERAL DISCUSSION

Our review suggests urban agriculture is rarely incorporated into undergraduate biology education, but there are significant opportunities for doing so that could enhance educational offerings. Our review of major programs found that although some biology‐related programs have courses focused on food systems and urban challenges, these courses are rare and only one mentions urban agriculture. In a survey of the AASHE STARS database, we found that a potential tool for urban agriculture, campus gardens, is common in U.S. HEIs, but few seem to be used in undergraduate biology courses. Our case studies of five HEIs engaging with this issue highlight innovative curricular and co‐curricular programming, but each identifies significant opportunities for further implementation. Together this work suggests that incorporating urban agriculture into undergraduate biology can help programs develop field and community engagement experiences, facilitating the implementation of Vision and Change recommendations.

Implementation of pedagogical innovations often faces significant barriers due to several factors including time limitations (e.g., for developing curricula and community partnerships), lack of professional rewards, and other logistical constraints on instructors. Here we make four recommendations for how to incorporate urban agriculture into biology curricula with an eye toward these barriers.

First, we recommend, where possible, building, developing, and using campus gardens for biology courses. We found that 94% of HEIs submitting AASHE STARS reports had campus gardens but few reported using them in biology courses. Incorporating these gardens into courses could help lab instructors create inquiry‐based research experiences for large numbers of students, a central recommendation in the Vision and Change document. Linking biology courses to campus gardens could also have diverse benefits for the garden and the broader community. Although campus gardens have the potential to provide many social and environmental benefits, some gardens fail or fall into disuse (Marsh et al., 2020). Main constraints include lack of staffing, consistent student support, and institutional financing. Creating biology labs and research projects based in a campus garden could help justify reallocation of departmental and college support from more traditional labs to the garden.

Second, we recommend that biology faculty develop research programs in urban agriculture and connect these programs to courses. Research‐based and student‐centered instruction can help transform education away from fact memorization and toward the development of skills for scientific inquiry and collaborative work (AAAS, 2011). Although the use of research‐based instruction is increasing in undergraduate life sciences education, many courses still rely primarily on lectures and exams focused on memorization of disciplinary content (AAAS, 2019). A promising approach to engage a large number and more diverse range of students in research is through course‐based undergraduate research experiences (CURE) (Auchincloss et al., 2014; Elgin et al., 2016; Hernandez et al., 2021). If faculty can initiate CUREs that align with their existing research efforts, it can allow them to benefit from reward structures that encourage research productivity (Smith et al., 2021). There are many opportunities for impactful research on applied urban agriculture and urban agroecology that can connect scholars, urban growers, and communities in mutually beneficial partnerships (Nicklay et al., 2020). In addition, urban agriculture can provide equally valuable opportunities for basic research on central topics in ecology, including biodiversity, biogeochemical cycling, abiotic controls on production, and human‐mediated feedbacks.

Third, we recommend that biology educators work with community engagement experts to build collaborations with community groups working in the local food system. Community engagement offices are becoming more common in HEIs, a movement inspired by a growing awareness that students are motivated by helping others. Benefits of community‐engaged learning that goes beyond developing scientific competencies include improving students’ communication, critical thinking, and writing skills (Astin et al., 2000; Eyler & Giles, 1999; Gallini & Moely, 2003; Vogelgesang, & Astin, 2000). Furthermore, community‐engaged learning gives students an opportunity to work with people from diverse backgrounds and gain experience with conflict resolution (Moely et al., 2002; Simons & Cleary, 2006). Finally, community‐engaged learning helps students feel connected to their community and take responsibility in addressing social justice issues (Munter, 2002). As a first step in developing a curriculum that centers community engagement, biology educators could engage with the Science Education for New Civic Engagements and Responsibilities (SENCER) program, which helps faculty develop courses connected to real‐world challenges. Assessments have found that civic engagement emphasized in SENCER has increased student academic confidence and interest in science. Further engagement could involve faculty connecting courses to urban agriculture organizations that are active in most U.S. cities. Organizations such as Infinite Zion Farms in Orlando are interested in deepening relationships with university partners. If done with care, these partnerships could provide impactful learning experiences for students while providing resources and connections for partner organizations. University support for community urban agriculture projects could help expand urban food production and increase urban food system resilience in a time of increasing uncertainty (Yan et al., 2022).

Creating effective, reciprocal relationships between academia and community organizations requires careful planning (Jordaan & Mennega, 2021). Academics engaging in these partnerships should ensure that collaborations benefit rather than burden community partners (Bringle & Hatcher, 2002). Situating community partners as co‐educators, for example, gives community members an opportunity to share their expertise with students to enhance their learning (for a more extensive discussion of this important issue, see Jordaan & Mennega, 2021). On‐campus community engagement offices can be useful for helping to best navigate these and other potential challenges.

Finally, we recommend university personnel and community partners connect to networks for developing and sharing curricular ideas, creating cross‐site experiments, and connecting courses to community engagement. One opportunity is through our newly formed Training Undergraduate Biologists using Urban Agriculture (TUBA) network (www.tuba‐rcn.org).

Undergraduate biology education needs to increase engagement for diverse student audiences to reduce attrition and in turn help generate scholars and professionals ready to address the complex challenges associated with rapid global change and growing social inequalities. Urban agriculture as a theme could increase student engagement with biology education by highlighting pressing contemporary challenges, increasing opportunities for local field experiences, and creating new relationships with community organizations. Implementing our recommendations could help urban agriculture become common in undergraduate biology. If done with care, this integration could help students make positive community contributions within the context of required coursework, and help instructors feel a greater sense of accomplishment in this era of change and uncertainty.

## CONFLICT OF INTEREST

The authors have no financial or nonfinancial interests that are directly or indirectly related to the work submitted for publication.

## AUTHOR CONTRIBUTIONS

**Adam D. Kay:** Conceptualization (equal); Data curation (equal); Formal analysis (equal); Funding acquisition (equal); Investigation (equal); Methodology (equal); Project administration (equal); Writing – original draft (lead); Writing – review & editing (lead). **Eric J. Chapman:** Conceptualization (equal); Project administration (equal); Writing – review & editing (equal). **Jelagat D. Cheruiyot:** Conceptualization (equal); Project administration (equal); Writing – review & editing (equal). **Sue Lowery:** Conceptualization (equal); Project administration (equal); Writing – review & editing (equal). **Susan R. Singer:** Conceptualization (equal); Project administration (equal); Writing – review & editing (equal). **Gaston Small:** Data curation (equal); Methodology (equal); Writing – review & editing (equal). **Anne M. Stone:** Conceptualization (equal); Project administration (equal); Writing – review & editing (equal). **Ray Warthen:** Conceptualization (equal); Project administration (equal); Writing – review & editing (equal). **Wendy Westbroek:** Conceptualization (equal); Project administration (equal); Writing – review & editing (equal).

## Supporting information

Tables S1‐S2

## Data Availability

All data presented in this paper has been uploaded to Dryad (https://doi.org/10.5061/dryad.zw3r22898).
