# Supplementary material for: Potential for urban agriculture to support accessible and impactful undergraduate biology education
Source: Ecol Evol. 2022 Mar 14;12(3):e8721. doi: 10.1002/ece3.8721 (PMC8928874; doi:10.1002/ece3.8721)
Supplement: Supplementary file 1 — Tables S1‐S2 [file ECE3-12-e8721-s001.doc]

**Table S1A.** Institution-specific information from course descriptions in Biology-related departments in 20 top-ranked research HEIs. Data represent the number of courses for which select terms are mentioned in course titles or descriptions. “Field” mentions were filtered to include only courses that referenced field trips or field-based courses. “Communit” OR “service” OR “experientia” mentions were filtered to include only courses with community-engaged learning opportunities. “Food” OR “agricultur” OR “agro” mentions were filtered to include only courses related to food systems. “Urban” mentions were filtered to include only courses that focused on urban environments.

| **Institution** | **Department** | **Search term(s)** | | | | | | | |
| --- | --- | --- | --- | --- | --- | --- | --- | --- | --- |
|  |  | | “field” | “communit” OR “service” OR “experientia” | |  | “food” OR “agricultur” OR “agro” | “urban” | “urban agriculture” |
| Brown University | Biology | | 9 | | 0 |  | 4 | 0 | 0 |
| California Institute of Technology | Biology | | 1 | | 0 |  | 1 | 0 | 0 |
| Columbia University | Biological Sciences | | 8 | | 1 |  | 3 | 0 | 0 |
| Cornell University | Animal Physiology; Biochemistry; Computational Biology; Ecology and Evolutionary Biology; General Biology; Genetics, Genomics and Development; Human Nutrition; Insect Biology; Marine Biology; Microbiology; Molecular and Cell Biology; Neurobiology and Behavior; Plant Biology; Systematics and Biotic Diversity | | 34 | | 1 |  | 10 | 2 | 1 |
| Emory University | Biology | | 8 | | 0 |  | 1 | 0 | 0 |
| Georgia Institute of Technology | Biological Sciences | | 1 | | 1 |  | 0 | 0 | 0 |
| Johns Hopkins University | Biology | | 1 | | 0 |  | 0 | 0 | 0 |
| Massachusetts Institute of Technology | Biology | | 0 | | 0 |  | 0 | 0 | 0 |
| Michigan State University | Zoology, Biochemistry and Molecular Biology, Plant Biology | | 8 | | 0 |  | 0 | 0 | 0 |
| Northwestern University | Biological Sciences | | 1 | | 0 |  | 0 | 0 | 0 |
| Princeton University | Ecology & Evolutionary Biology, Molecular Biology | | 7 | | 0 |  | 2 | 0 | 0 |
| Purdue University | Biological Sciences | | 3 | | 1 |  | 3 | 0 | 0 |
| Stanford University | Biology | | 14 | | 1 |  | 1 | 0 | 0 |
| Texas A & M University | Biology, Microbiology, Zoology | | 1 | | 0 |  | 0 | 0 | 0 |
| University of California – Berkeley | Integrative Biology, Molecular and Cell Biology, Plant and Microbial Biology | | 24 | | 0 |  | 11 | 0 | 0 |
| University of California - Los Angeles | Ecology and Evolutionary Biology; Bioinformatics; Molecular, Cell, and Developmental Biology; Microbiology, Immunology, and Molecular Genetics | | 34 | | 3 |  | 2 | 0 | 0 |
| University of Michigan | Ecology and Evolutionary Biology; Molecular, Cellular, and Developmental Biology | | 14 | | 1 |  | 7 | 1 | 0 |
| University of Virginia | Biology | | 12 | | 0 |  | 2 | 0 | 0 |
| University of Washington | Biology | | 21 | | 0 |  | 2 | 0 | 0 |
| Yale University | Biology; Ecology and Evolutionary Biology; Molecular, Cellular, and Developmental Biology | | 9 | | 0 |  | 2 | 0 | 0 |

**Table S1B.** Institution-specific information from course descriptions in Biology-related departments in 20 top-ranked liberal arts HEIs. Data represent the number of courses for which select terms are mentioned in course titles or descriptions. “Field” mentions were filtered depending on whether it referenced to field trips or courses. “Communit” OR “service” OR “experientia” mentions were filtered depending on whether they referred to community-engaged learning opportunities. “Food” OR “agricultur” OR “agro” mentions were filtered depending on whether they were related to food systems. “Urban” mentions were filtered depending on whether they referred to urban environments

| **Institution** | **Department** | **Search term(s)** | | | | | |
| --- | --- | --- | --- | --- | --- | --- | --- |
|  |  | “field” (related to field experiences for students) | “communit” OR “service” OR “experientia” (related to community-engaged learning) |  | “food” OR “agricultur” OR “agro” (related to food systems) | “urban” (related to urban systems) | “urban agriculture” |
| Amherst College | Biology | 3 | 0 |  | 1 | 0 | 0 |
| Barnard  College | Biology | 1 | 0 |  | 0 | 0 | 0 |
| Bowdoin College | Biology | 4 | 0 |  | 1 | 0 | 0 |
| Carleton College | Biology | 2 | 1 |  | 2 | 0 | 0 |
| Claremont McKenna College | Biology | 9 | 0 |  | 0 | 0 | 0 |
| Colby  College | Biology | 7 | 0 |  | 3 | 0 | 0 |
| Colgate  University | Biology | 7 | 0 |  | 0 | 0 | 0 |
| Davidson  College | Biology | 9 | 0 |  | 0 | 1 | 0 |
| Grinnell  College | Biology | 7 | 0 |  | 0 | 0 | 0 |
| Hamilton  College | Biology | 3 | 0 |  | 4 | 1 | 0 |
| Haverford  College | Biology | 0 | 0 |  | 1 | 0 | 0 |
| Middlebury  College | Biology | 11 | 1 |  | 1 | 0 | 0 |
| Pomona College | Biology | 8 | 0 |  | 0 | 0 | 0 |
| Smith  College | Biological Sciences | 8 | 0 |  | 1 | 1 | 0 |
| Swarthmore College | Biology | 5 | 1 |  | 1 | 0 | 0 |
| University of Richmond | Biology | 2 | 0 |  | 0 | 1 | 0 |
| Washington and Lee University | Biology | 4 | 1 |  | 2 | 0 | 0 |
| Wellesley College | Biological Sciences | 4 | 0 |  | 5 | 1 | 0 |
| Wesleyan University | Biology | 5 | 0 |  | 7 | 1 | 0 |
| Williams College | Biology | 5 | 0 |  | 2 | 1 | 0 |

**Table S2.** Questions from American Association for Sustainability in Higher Education (AASHE) Sustainability Tracking and Assessment System (STARS) reports that were analyzed for integration of campus farms/gardens in teaching or research.

| **Section of report** | **Question** |
| --- | --- |
| EN-3: Student Life | Does the institution have gardens, farms, community supported agriculture (CSA) or fishery programs, and/or urban agriculture projects where students are able to gain experience in organic agriculture and sustainable food systems? |
| EN-3: Student Life | A brief description of the gardens, farms, community support agriculture (CSA) or fishery programs, and/or urban agriculture projects |
| EN-3: Student Life | The website URL where information about the gardens, farms or agriculture projects is available |
| AC-8: Campus as a Living Laboratory | Is the institution utilizing its campus as a living laboratory for multidisciplinary student learning and applied research in relation to Food & Dining? |
| AC-8: Campus as a Living Laboratory | A brief description of the student/faculty projects and how they contribute to understanding campus sustainability challenges or advancing sustainability on campus in relation to Food & Dining |
| AC-8: Campus as a Living Laboratory | Is the institution utilizing its campus as a living laboratory for multidisciplinary student learning and applied research in relation to Grounds? |
| AC-8: Campus as a Living Laboratory | A brief description of the student/faculty projects and how they contribute to understanding campus sustainability challenges or advancing sustainability on campus in relation to Grounds |
